# CD69 expression potentially predicts response to bendamustine and its modulation by ibrutinib or idelalisib enhances cytotoxic effect in chronic lymphocytic leukemia

**DOI:** 10.18632/oncotarget.6685

**Published:** 2015-12-19

**Authors:** Arnau Montraveta, Eriong Lee-Vergés, Jocabed Roldán, Laura Jiménez, Sandra Cabezas, Guillem Clot, Magda Pinyol, Sílvia Xargay-Torrent, Laia Rosich, Cristina Arimany-Nardí, Marta Aymerich, Neus Villamor, Armando López-Guillermo, Patricia Pérez-Galán, Gaël Roué, Marçal Pastor-Anglada, Elías Campo, Mónica López-Guerra, Dolors Colomer

**Affiliations:** ^1^ Institut d'Investigacions Biomèdiques August Pi i Sunyer (IDIBAPS), Barcelona, Spain; ^2^ Departament de Bioquímica i Biologia Molecular, Institut de Biomedicina, Universitat de Barcelona and Oncology Program, CIBEREHD, Barcelona, Spain; ^3^ Hematopathology Unit, Hospital Clinic, IDIBAPS, Barcelona, Spain; ^4^ Hematology Department, Hospital Clinic, IDIBAPS, Barcelona, Spain

**Keywords:** bendamustine, CD69, ibrutinib, idelalisib, chronic lymphocytic leukemia

## Abstract

Clinical responses to bendamustine in chronic lymphocytic leukemia (CLL) are highly heterogeneous and no specific markers to predict sensitivity to this drug have been reported. In order to identify biomarkers of response, we analyzed the *in vitro* activity of bendamustine and the gene expression profile in primary CLL cells. We observed that mRNA expression of *CD69* (CD69) and *ITGAM* (CD11b) constitute the most powerful predictor of response to bendamustine. When we interrogated the predictive value of the corresponding cell surface proteins, the expression of the activation marker CD69 was the most reliable predictor of sensitivity to bendamustine. Importantly, a multivariate analysis revealed that the predictive value of CD69 expression was independent from other clinico-biological CLL features. We also showed that when CLL cells were co-cultured with distinct subtypes of stromal cells, an upregulation of CD69 was accompanied by a reduced sensitivity to bendamustine. In agreement with this, tumor cells derived from lymphoid tumor niches harbored higher CD69 expression and were less sensitive to bendamustine than their peripheral blood counterparts. Furthermore, pretreatment of CD69 ^high^ CLL cases with the B-cell receptor (BCR) pathway inhibitors ibrutinib and idelalisib decreased CD69 levels and enhanced bendamustine cytotoxic effect. Collectively, our findings indicate that CD69 could be a predictor of bendamustine response in CLL patients and the combination of clinically-tested BCR signaling inhibitors with bendamustine may represent a promising strategy for bendamustine low responsive CLL cases.

## INTRODUCTION

Chronic lymphocytic leukemia (CLL) is characterized by the proliferation and progressive accumulation of mature clonal B lymphocytes in the peripheral blood, bone marrow, and lymphoid tissues. The clinical course of the disease is highly heterogeneous, with some patients requiring early treatment because of disease progression while others have an indolent course that does not affect life expectancy [1, 2]. Several biological features, including immunoglobulin heavy chain variable region (*IGHV*) mutational status, cytogenetic abnormalities and the expression of ZAP-70, CD38 or CD49d, have been related to patient outcome [1]. In recent years, in addition to the known alterations in DNA-repair genes (*TP53* and *ATM*), next generation sequencing has identified new somatic mutations (*NOTCH1, SF3B1, BIRC3, POT1, MYD88*) with predicted functional relevance in CLL [3–6]. Despite their relative low frequency, these new mutations could in part explain CLL heterogeneity and help in identifying clinically relevant groups of patients [4, 5, 7] and new targeted therapies in this entity [8, 9].

The standard of care for CLL is a cytotoxic therapy that includes fludarabine plus cyclophosphamide with the monoclonal antibody rituximab (FCR) [10]. Bendamustine is a well-tolerated and less toxic agent that has emerged as a feasible therapy for elderly and non-fit CLL patients [11]. Several trials have shown its clinical efficacy in previously untreated and refractory CLL patients being the combination of bendamustine with rituximab one of the first-line treatments for elderly patients with CLL lacking 17p deletion [12–15]. Nowadays, *in vitro* results and ongoing clinical trials have explored the combination of bendamustine with new generation monoclonal antibodies [16, 17] and novel targeted agents[18–22].

Bendamustine is a bifunctional alkylating agent that contains a nitrogen mustard group and a benzimidazole nucleous, combining the properties of an alkylator and a purine analogue. These structural characteristics confer the compound a unique mechanism of action, with only partial cross-resistance to other alkylating agents and antimetabolites [23]. Differences have been observed in regard to its effects on DNA repair and cell cycle progression. Moreover, bendamustine engages cell death through both apoptotic and non-apoptotic pathways, thereby retaining activity even in cells with dysfunctional apoptotic machinery [24]. Recently, it has been reported that some membrane transporters might also contribute to the cytotoxic effect of bendamustine [25, 26].

Herein, a high-throughput gene expression analysis allows us to identify putative biomarkers that could predict clinical response of CLL patients to bendamustine. In addition, we propose rational drug combinations to overcome resistance to bendamustine.

## RESULTS

### Correlation of gene expression profile and sensitivity to bendamustine

To evaluate sensitivity to bendamustine, primary cells from 38 untreated CLL cases were incubated with the drug at the physiological dose of 25 μM and cytotoxicity was determined after 24 hours by double staining with Annexin-V/propidium iodide (PI). As expected, the apoptosis induced by bendamustine was highly heterogeneous, ranging from 4.9 to 79.2% (Supplemental Table S1). In agreement with clinical data of CLL patients treated with bendamustine, the only CLL case carrying a 17p deletion (CLL 32) was resistant to bendamustine.

In order to find potential markers of response to bendamustine, we carried out a gene expression profiling of these 38 CLL cases and analyzed the differential expressed genes between the 10 most sensitive cases, all of them with a cytotoxicity higher than 42%, and the 10 most resistant cases, with response to bendamustine of less than 18%. To identify the differentially expressed genes (DEGs) between sensitive and resistant samples, we applied a supervised analysis using a Rank Products through the Multiexperiment Viewer Platform (TM4-MeV) which is based on a two-class unpaired analysis, using a false discovery rate (FDR) below 0.01. Next, we discarded those genes with a fold-change below 1.5. This supervised analysis identified 414 DEGs out of 19975, being 238 significantly down-regulated and 176 up-regulated in the bendamustine resistant group (Supplemental Table S2).

**Figure 1:**
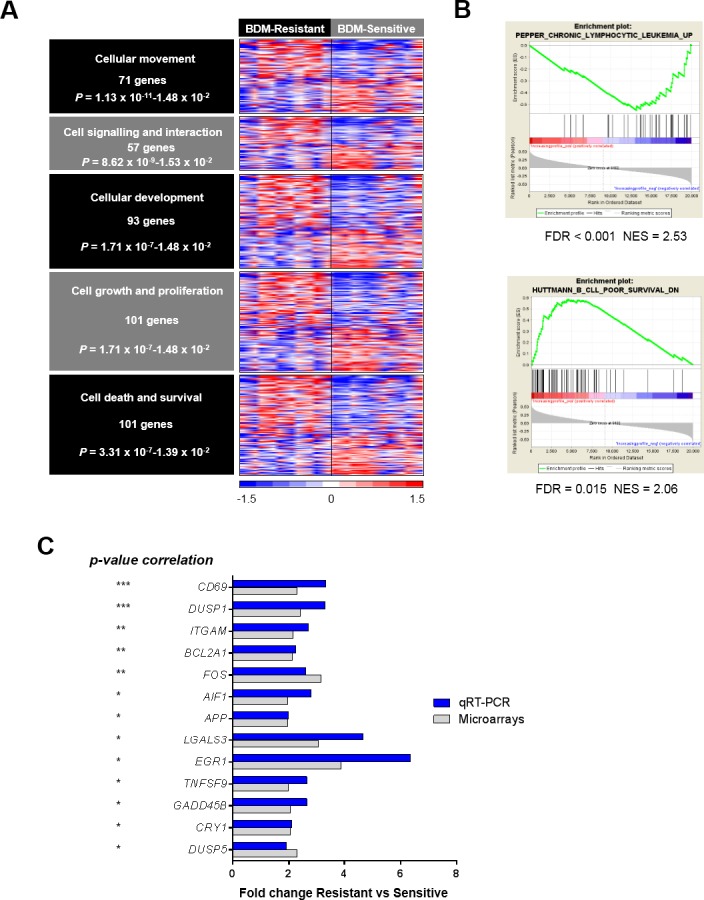
Gene expression profile of bendamustine-resistant and -sensitive cases **A**. Top biological enriched functions (IPA analysis) with the DEGs (RP analysis, FDR < 0.01) between the 10 most resistant and the 10 most sensitive cases to bendamustine (fold-change > 1.5). Relative gene expression levels are color-coded as indicated at the bottom legend. **B**. GSEA enrichment plots of the CLL signatures showing a correlation with sensitivity to bendamustine. NES, normalized enrichment score. **C**. Validation of the 13 DEGs by qRT-PCR in a large cohort of cases (*p-value* indicated at left). Fold-changes of these genes are displayed for both qRT-PCR and microarray analysis in the resistant and sensitive groups. *, *P < 0.05;* **, P < 0.01; ***, *P < 0.001.*

To gain insights into the biologic meaning of the differential expression profile between bendamustine-sensitive and -resistant CLL cases, we conducted a functional enrichment analysis using the Ingenuity Pathway analysis (IPA) application using a specific filter for human B lymphocytes and lymphoma/leukemia cells. As shown in Figure 1A, the top biological processes enriched with the DEGs were cellular movement (71 genes), cell-to-cell signaling and interaction (57 genes), cellular development (93 genes), cellular growth and proliferation (101 genes) and cell death and survival (100 genes). The complete list of genes is shown in Supplemental Table S3.

We performed a Gene Set Enrichment Analysis (GSEA) using the C2 (curated gene sets) collection from the Molecular Signature Database v2.5, with an increasing profile analysis, 1000 permutations of gene sets and a weighted metric. Thus, we identified two gene signatures related to CLL features whose gene expression correlated with response to bendamustine (gene sets with an FDR below 0.05 were considered). Bendamustine-resistant samples were enriched in genes previously found to be up-regulated in CD38^+^ cells [27] whereas bendamustine-sensitive cases displayed up-regulation of genes down-regulated in ZAP-70^+^ CD38^+^ CLL cases (Figure 1B) [28].

### Validation of DEGs between bendamustine-resistant and -sensitive CLL

To validate the gene expression profile results, we selected a set of 46 DEGs from the biological functions encountered by IPA whose fold-change was above 2. These genes were analyzed by qRT-PCR using the Fluidigm platform in a cohort of 77 CLL cases. Thirteen genes from the selected list displayed an inverse correlation with response to bendamustine (Supplemental Table 4). *CD69* and *DUSP1* were the genes with the best significant correlation with bendamustine response (*P* < 0.001), and a significant correlation of *P <* 0.01 was observed for the expression of *ITGAM*, *BCL2A1 and FOS.* All validated genes had a fold-change > 2 between the resistant and sensitive groups defined as above, with a similar trend as observed in the microarray analysis (Figure 1C).

### CD69 expression as a predictor of bendamustine response in CLL

**Figure 2:**
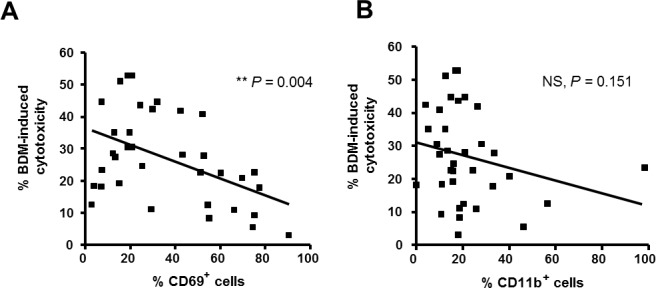
Association of CD69 surface levels with response to bendamustine Basal percentages of CD69^+^ **A**. and CD11b^+^ **B**. CLL cells were quantified by flow cytometry in 35 CLL cases and correlation with response rates to bendamustine 25 μM at 24 hours was analyzed. BDM, bendamustine; **, *P < 0.01; NS, not significant.*

With the aim to obtain a predictive gene signature of response to bendamustine, we used the gene expression data validated by qRT-PCR to estimate the Root Mean Squared Error (RMSE) metric of different combination of genes as described in *Materials and Methods*. In order to obtain the combination of validated genes that best predicts the response to bendamustine, a linear regression model was fitted for each combination of one to six genes. The best cytotoxicity predictor was a signature composed of two genes, the activation marker *CD69* and the integrin *ITGAM,* which had the lowest RMSE (14.976) (Table 2A). The most powerful one-variable predictor was *ITGAM* (RMSE = 15.451), followed by *CD69* (RMSE = 15.849) (Table 2B). *CD69* and *ITGAM* codify for CD69 and CD11b molecules respectively, both expressed mainly on the cell surface. To further validate our results, we quantified CD69 and CD11b protein levels by flow cytometry in primary cells from 35 of the CLL cases studied by qRT-PCR. Then, a second analysis to check the predictive value of CD69 and CD11b protein levels was performed. CD69 levels (*P* = 0.004) showed a correlation with sensitivity to the drug whereas no correlation was observed with CD11b protein expression (Figure 2). These results prompted us to focus on the single expression of CD69 for further study. Relative *CD69* mRNA expression levels using a Universal Human Reference RNA as a calibrator and expression of CD69 by flow cytometry are showed in Supplemental Table 1.

**Table 1:** Clinical and biological characteristics of CLL cases

Variable	Category	CLL cases (*n* = 80)
**Age at diagnosis**		
	**Median (range)**	58.7 (37-80)
**Gender (F/M)**		
	**Female**	22 (27.5%)
	**Male**	58 (72.5%)
**Percentage of tumor cells**		
	**Median (range)**	97 (64-99)
**Rai stage**		
	**0**	38 (47.50%)
	**I**	12 (15%)
	**II**	18 (22.5%)
	**III**	5 (6.25%)
	**IV**	7 (8.75%)
**Binet stage**		
	**A**	51 (63.75%)
	**B**	18 (22.5%)
	**C**	11 (13.75%)
**Previous treatment**		
	**Untreated**	68 (85%)
	**Treated**	12 (15%)
**IGVH status**		
	**Mutated**	46 (57.5%)
	**Unmutated**	33 (41.25%)
	**Not evaluated**	1 (1.25%)
**ZAP-70 expression**		
	**ZAP-70**^−^	57 (71.25%)
	**ZAP-70**^+^	22 (27.5%)
	**Not evaluated**	1 (1.25%)
**CD38 expression**		
	**CD38**^−^	61 (76.25%)
	**CD38**^+^	18 (22.5%)
	**Not evaluated**	1 (1.25%)
**CD49d expression**		
	**CD49d**^−^	15 (18.75%)
	**CD49d**^+^	46 (57.5%)
	**Not evaluated**	19 (23.75%)
**Cytogenetics**		
	**Deletion of 17p**	2 (2.5%)
	**Trisomy 12**	8 (10%)
	**Deletion of 13q**	44 (55%)
	**Deletion of 11q**	10 (12.5%)
	**Normal**	20 (25%)
	**Not evaluated**	1 (1.25%)
**Recurrent mutations**		
	***ATM***	7 (8.75%)
	***SF3B1***	5 (6.25%)
	***NOTCH1***	4 (5%)
	***MYD88***	2 (2.5%)
	***TP53***	4 (5%)

**Table 2:** Potential predictor signatures for response to bendamustine obtained by performing an RMSE analysis on mRNA data

A: List of the first 10 predictor signatures		
Number of variables	Variables	RMSE
2	*CD69, ITGAM*	14.976
4	*CD69, GADD45B, ITGAM,TNFSF9*	14.997
3	*CD69, GADD45B, ITGAM*	15.041
3	*AIF1, CD69, ITGAM*	15.099
3	*CD69,ITGAM,FOS*	15.157
4	*AIF1,CD69,GADD45B,ITGAM*	15.196
3	*CD69,ITGAM,TNFSF9*	15.212
3	*BCL2A1,CD69,ITGAM*	15.213
4	*AIF1,CD69,ITGAM,FOS*	15.258
3	*CD69,DUSP1,ITGAM*	15.282
B: List of the first 10 predictor genes		
**Number of variables**	**Variables**	**RMSE**
1	*ITGAM*	15.451
1	*CD69*	15.849
1	*DUSP1*	16.052
1	*TNFSF9*	16.144
1	*AIF1*	16.291
1	*FOS*	16.352
1	*BCL2A1*	16.514
1	*GADD45B*	16.568
1	*DUSP5*	16.571
1	*EGR1*	16.664

### CD69 as an independent marker of response to bendamustine in CLL

To confirm the potential value of CD69 as a new biomarker of bendamustine response in CLL, we analyzed whether its predictive significance was independent from the main clinical and biological characteristics of CLL cases included in the study (*n* = 77). Through a Mann Whitney U Test we observed that, besides high CD69 mRNA expression (*P* = 0.0004), prior treatment history (*P* = 0.0189) was also associated with low response to bendamustine. However, a multivariate analysis including Rai/Binet stage, previous treatment, presence of cytogenetic alterations (11qdel, 17pdel, 13qdel and trisomy 12), positivity for the prognostic factors CD49d, CD38, ZAP-70, *IGHV* mutational status and presence of recurrent mutations (*ATM, TP53, NOTCH1, SF3B1, MYD88 and BIRC3*), revealed that CD69 mRNA level was the only variable with independent predictive value of bendamustine *in vitro* cytotoxicity. These results support the predictive value of CD69 expression as an independent marker of bendamustine *in vitro* response in CLL.

### CLL microenvironment induces CD69 expression and reduces sensitivity to bendamustine

**Figure 3:**
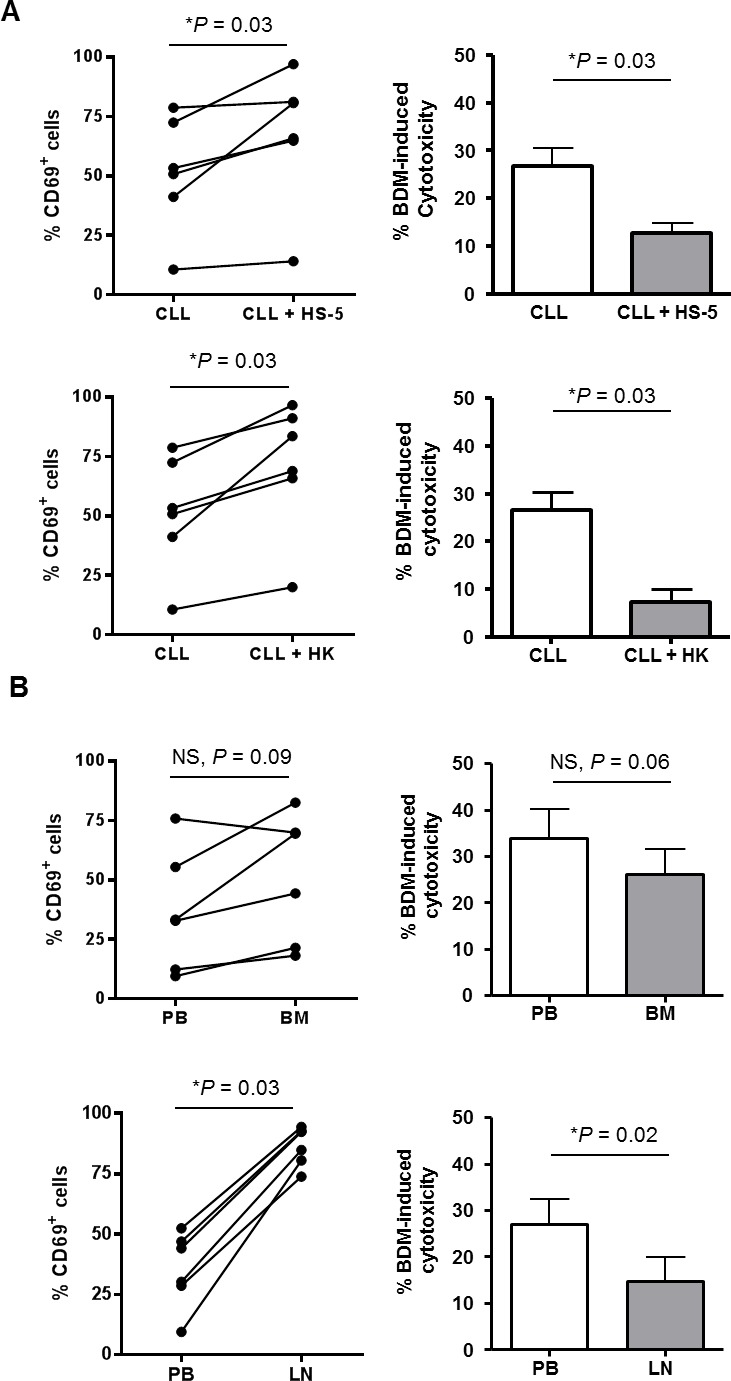
CD69 as a marker of bendamustine-resistance in lymphoid tissue compartments **A**. Percentage of CD69^+^ CLL cells was determined after 24 hours of co-culture with HS-5 or HK cell lines (*n* = 6; left panels). At this point, bendamustine 25 μM was added and cytotoxicity was quantified 24 hours later. Cytotoxicity is referred to the untreated control in each culture condition. Bars represent the mean±SEM of all samples analyzed (right panels). *, *P < 0.05.* **B**. Basal protein levels of CD69 were determined in bone marrow (BM) and lymph node (LN) CLL samples (*n* = 6; left panels). Cells were exposed to bendamustine 25 μM for 24 hours and cytotoxicity was analyzed by triple labelling with anti-CD19 antibody, Annexin-V and PI. Cytotoxicity in each tissue compartment is referred to cytotoxicity induced in PB cells. Bars represent the mean±SEM of all samples analyzed (right panels). BDM, bendamustine.

**Figure 4:**
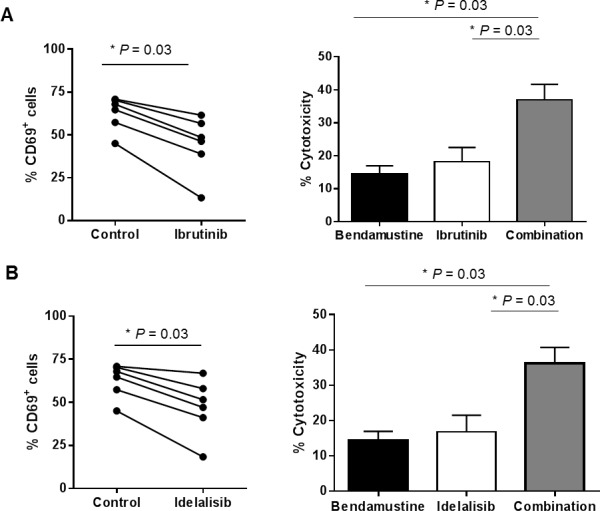
BCR pathway inhibitors as a strategy to sensitize CD69^*high*^ CLL cells to bendamustine Cells from CD69^*high*^ CLL cases were preincubated for 24 hours with ibrutinib 1 μM **A**. or idelalisib 0.5 μM **B**., followed by a 24-hour incubation with bendamustine 25 μM. Percentage of both CD69^+^ (left panels) and Annexin-V^+^ cells (right panels) were quantified at the end point. Cytotoxicity values are referred to the untreated control. Bars represent the mean±SEM of all samples analyzed (*n* = 6) *, *P < 0.05*.

Microenvironment interactions in lymphoid tissues are reported to protect tumoral CLL cells against the action of conventional chemotherapeutic drugs [29, 30]. In particular, CD69 has been shown to be up-regulated on CLL cells in the tissue microenvironment, both in bone marrow (BM) and lymph node (LN) [31]. With the hypothesis that CD69 could be involved in the development of the resistance to bendamustine mediated by the microenvironment, we co-cultured primary CLL cells with the human bone marrow-derived stroma cell line HS-5 and the human follicular dendritic cell-like cell line HK. As previously reported [20, 22, 32], the presence of microenvironment-mimicking cells significantly reduced bendamustine-induced apoptosis (HS-5: *P* = 0.03; HK: *P* = 0.03; Figure 4A). Importantly, we showed that CD69 protein levels were increased when CLL cells were co-cultured both with HS-5 and HK cells (31.4% and 38.7% of increase, respectively; Figure 3A) in comparison to monocultured cells. We also compared CD69 surface levels and bendamustine response of tumor lymphocytes derived from the three typical anatomic CLL compartments: peripheral blood (PB), BM and LN. For this purpose, we treated 6 paired PB- and BM-derived CLL cells and 6 paired PB- and LN-derived samples with bendamustine 25 μM for 24 hours. As shown in Figure 3B, BM-resident CLL cells, although not significantly, were less sensitive than their respective PB counterparts (*P =* 0.06) whereas this trend was significant in LN-resident cells (*P* = 0.03). Accordingly, we found a higher CD69 expression in tissue-resident CLL cells compared to PB samples (mean increase of 40% in BM and 182% in LN) (Figure 3B). All these results suggest that the activation marker CD69 could exert a role in the resistance to bendamustine mediated by the microenvironment.

### BCR pathway inhibitors decrease CD69 levels and sensitize CD69^*high*^ CLL cells to bendamustine

The BCR pathway inhibitors ibrutinib and idelalisib are showing good clinical activity in CLL, in part by reducing the degree of cellular activation [33, 34]. Particularly, it has been reported that ibrutinib is able to down-modulate CD69 surface levels in the clinical setting [34]. Thus, we hypothesized that BCR inhibitors could have a role in sensitizing CD69*^high^* samples to bendamustine. To prove this, we preincubated CLL cells expressing high levels of CD69 (> 30%) with ibrutinib 1 μM or idelalisib 0.5 μM for 24 hours before adding bendamustine for 24 additional hours. Both ibrutinib and idelalisib decreased CD69 surface protein expression at 24 hours (data not shown) and more markedly at 48 hours (Figure 4). Importantly, the modulation of this activation marker was accompanied by a collaborative antitumor effect between both drugs in terms of cytotoxicity. In this set of bendamustine resistant CLL cases (14.4±2.5% cytotoxicity), ibrutinib induced 18.15±4.7% cytotoxicity whereas the combination of both drugs had a more marked antitumor effect (36.9±5.2%) (Figure 4A). Similarly, idelalisib (16.8±5.1%) also potentiated bendamustine activity (36.1±4.9%) (Figure 4B). These results indicate that BCR inhibitors may be useful to sensitize CD69*^high^* CLL cases to bendamustine.

## DISCUSSION

Patients with CLL are generally managed with a ‘watch and wait’ strategy until an indication for treatment emerges [10, 35]. The initial standard therapy for patients without del(17)(p13.1) is chemoimmunotherapy (fludarabine plus cyclophosphamide, bendamustine, or chlorambucil) and anti-CD20 antibody (rituximab, ofatumumab, or obinutuzumab) that varies based on regimen and patient status [11]. Bendamustine as monotherapy or in combination with other chemotherapeutic agents is currently indicated for the treatment of elderly and less fit CLL patients lacking del(17)(p13.1) [12, 15]. The primary action of bendamustine is the activation of a DNA damage stress response and the inhibition of mitotic checkpoints, leading to cell death *via* mitochondrial apoptosis or mitotic catastrophe induction [36–38].

The response of CLL cells to bendamustine shows a marked variability and the molecular mechanisms related to bendamustine resistance remain largely undefined. Although it has been observed a lower response to bendamustine in cases carrying 17p/p53 alterations, this genetic abnormality does not fully account for heterogeneity in responsiveness to treatment. Herein, we have performed a gene expression high-throughput analysis with the aim of identifying putative biomarkers that could predict the clinical response of CLL to bendamustine. Our results showed that the DEGs between the bendamustine-sensitive and -resistant cases belong to biological processes related to cell death and survival, cellular growth and proliferation. GSEA analysis revealed that genes overexpressed in CLL signatures associated with poor prognostic factors [27, 28] were similarly expressed in bendamustine-resistant cases. Among the DEGs, we found that the mRNA expression of the activation marker *CD69* and the integrin *ITGAM* were the most reliable predictors of resistance to bendamustine in CLL cells. However, at protein level only CD69 expression correlated with bendamustine-induced cytotoxicity. Moreover, a multivariate analysis revealed that CD69 expression was the only factor that predicts response to bendamustine independently from other biological and clinical CLL features, pointing out a functional role of this protein in the mechanisms of resistance to bendamustine.

CD69 is a type II integral membrane protein belonging to the C-type lectin family of surface receptors and is expressed in all bone marrow-derived cells, with the exception of erythrocytes [39]. CD69 is not found on resting circulating lymphocytes in humans, although *in vitro* cell activation showed rapid induction on human T and B lymphocytes This surface protein is commonly used as the marker of activated cells, most often in lymphocytes and natural killer cells [40]. Recently, it has also been described to be a key regulator of immune responses and one of the major regulators of lymphocyte migration, particularly at the mucosal sites [41]. In CLL, CD69 has been reported to be an independent prognostic marker that significantly correlates with poor clinical and biological prognostic factors such as the number of treatment lines, the mutational status of the *IGHV* genes, and the expression of CD38, ZAP-70 and CD49d [42, 43]. In our study, we confirmed the correlation between CD69 and CD38 expression and the association between previous treatment and bendamustine cytotoxicity *in vitro*. Of note, the GSEA analysis also showed that those CLL cases with high expression of CD69 and low response to bendamustine had a similar expression profile of CD38 and ZAP-70 positive CLL cells [27, 28].

Furthermore, our results provided evidence that microenvironment-driven resistance to bendamustine is accompanied by the up-regulation of CD69 in CLL cells considering both mesenchymal and dendritic cell co-culture systems. This cytotoxic protection by the microenvironment has been reported previously by our group [20, 22] and others [32, 44]. In line with these results, we observed that tissue-derived CLL cells had higher CD69 expression levels than their peripheral blood counterparts as previously reported [31, 45]. Accordingly, we demonstrated that both LN- and BM-derived CLL cells were less sensitive to bendamustine than their PB counterparts. On this basis, we postulate that the *in vivo* bendamustine sensitivity could be explained, at least partially, by the microenvironment-mediated induction of CD69 expression on CLL cells.

This hypothesis prompted us to test if CD69*^high^* CLL cells without p53 alterations (CD69 expression > 30%)[43] could be sensitized to bendamustine with some therapeutic strategy. In this context, it has been described that the BTK inhibitor ibrutinib was able to decrease CD69 surface levels on CLL cells *in vivo* [34]. We confirmed that ibrutinib is able to down-regulate CD69 levels and that bendamustine plus ibrutinib have a cooperative antitumor effect. These data agree with the promising results reported in clinical trials evaluating the safety and the efficacy of ibrutinib in combination with bendamustine-based regimens in relapsed CLL [18, 46]. As preclinical investigation of the combination of ibrutinib and rituximab resulted in an antagonistic effect [47], it is conceivable that bendamustine might play an important role in the potentiation of ibrutinib effect. Our results showed that the PI3Kδ inhibitor idelalisib also decreased CD69 levels and enhanced bendamustine-induced cytotoxicity in primary CLL cells. In this regard, there are ongoing clinical trials evaluating the combination of bendamustine-based regimens and idelalisib [48, 49]. We can hypothesize that the two approved targeted therapies in CLL cells, ibrutinib and idelalisib, although they differ considerably from each other, not only in target but also in mechanism of action, could facilitate bendamustine *in vivo* activity. By disruption of the microenvironment-supporting growth, both drugs result in the egress of the tumor cells from lymphoid tissues to peripheral blood, where microenvironment signals are much weaker and bendamustine might exert a greater effect. In this sense, experiments in mouse models have reported that bendamustine is mainly confined to the extracellular fluid and not extensively distributed to tissues [50]. Through this mechanism, CLL cells mobilized after treatment with ibrutinib could be sensitive to apoptotic-triggering drugs [19].

In summary, our results support a role of the activation marker CD69 in the resistance of CLL cells to bendamustine, suggesting that its surface levels could predict response of patients to this compound. Furthermore, we propose that bendamustine efficacy could be increased by its combination with the state-of-the-art BCR pathway inhibitors ibrutinib or idelalisib, as both drugs were able to decrease CD69 levels and therefore sensitize CLL cells to bendamustine. These combinations, already in clinical trials, could be a potential strategy to treat poor prognosis CD69*^high^* CLL cases as well as to contribute to the achievement of complete remissions during treatment with these targeted therapies.

## MATERIALS AND METHODS

### Isolation and culture of primary cells

Peripheral blood mononuclear cells (PBMCs) from 80 patients diagnosed with CLL according to the World Health Organization criteria [51] were used in this study. Clinical and biological data of these cases are summarized in Table 1 and detailed in Supplemental Table 1.

The *IGHV* gene mutational status was determined according to European Research Initiative on CLL (ERIC) guidelines [52]. Percentage of tumor cells (CD19+, CD5+) and expression of ZAP-70, CD38 and CD49d were determined by flow cytometry. The identification of cytogenetic aberrations involving 11q22-23 (*ATM*), 13q14 and 17p13 (*TP53*) deletions and trisomy 12 was done by fluorescence *in situ* hybridization (FISH). Recurrent mutations were obtained from previous whole genome/exome sequencing studies [4].

Primary cells were isolated from PB or BM samples by Ficoll-Paque sedimentation (GE-Healthcare). Primary cells from LN were obtained after squirting with RPMI 1640 (Life Technologies) culture medium using a fine needle. Samples were cryopreserved and stored within the Hematopathology collection of our institution registered at the Biobank from Hospital Clínic-IDIBAPS (R121004-094). The ethical approval for this project including the informed consent of the patients was granted following the guidelines of the Hospital Clínic Ethics Committee. Thawed cells were cultured in fresh RPMI 1640, supplemented with 10% fetal bovine serum (FBS), 2 mM glutamine and 50 μg/mL penicillin-streptomycin (Life technologies) and cultured in a humidified atmosphere at 37°C containing 5% carbon dioxide.

### Analysis of cytotoxicity

Primary CLL cells were incubated for 24 hours with a physiological dose of 25 μM bendamustine, kindly provided by Mundipharma. When indicated, CLL cells were pretreated for 24 hours with 0.5 μM of the PI3Kδ inhibitor idelalisib and 1 μM of the BTK inhibitor ibrutinib (Selleck Chemicals) prior to bendamustine addition. Cell viability was quantified by double staining with Annexin-V conjugated to fluorescein isothiocyanate (FITC) and PI (eBiosciences). For the comparative analysis of response in PB, BM and LN compartments, cells were triple-stained with phycoerythrin (PE)-conjugated anti-CD19 (Becton Dickinson), Annexin-V-FITC and PI. Labeled samples were analyzed on an Attune focusing acoustic cytometer (Life Technologies). Cytotoxicity (mean ± SEM) was calculated as the percentage of Annexin-V-positive cells in treated samples relative to the untreated ones.

### Gene expression analysis

CD19^+^ tumor CLL cells were purified from 38 CLL cases as previously reported [3]. Total RNA was isolated from these samples using TRIzol reagent (Life Technologies) according to manufacturer's instructions. RNA integrity was examined with the Bioanalyzer 2100 (Agilent Technologies) and only high quality samples were hybridized to Affymetrix GeneChip HT HG-U219 perfect-match-only (PM) Array Plate, following Affymetrix standard protocols. The Expression Console software (Affymetrix, Santa Clara, CA, USA) was used to get the summarized expression values by the Robust Multi-array Analysis (RMA). The raw data have been deposited in the Gene Expression Omnibus Database (accession number GSE68163).

DEGs were identified by using a Rank Products (RP) test through the Multiexperiment Viewer Platform (TM4-MeV), which is based on a two-class unpaired analysis as previously described [53], using a FDR below 0.01. Genes with a fold-change below 1.5 were discarded. The list of DEGs was used to conduct a functional enrichment analysis using the Ingenuity Pathway analysis (IPA)(Ingenuity^®^ Systems) and applying a specific filter for human B lymphocytes and lymphoma/leukemia cells.

Gene signatures with an expression pattern correlating with sensitivity to bendamustine were identified with GSEA version 2.0 (Broad Institute at MIT; http://www.broadinstitute.org/gsea/) using the C2 (curated gene sets) collection from the Molecular Signature Database v2.5. An increasing profile analysis with 1000 permutations of gene sets and a weighted metric was used. Gene sets with an FDR below 0.05 were considered.

### Quantitative real-time PCR

Total RNA was isolated as above from CLL cells, and complementary DNA was obtained using the Reverse Transcription Master Mix (Fluidigm Corporation). Samples were processed to Specific Target Amplification using the PreAmp Master Mix (Fluidigm Corporation) and the corresponding TaqMan Gene Expression Assays (Life Technologies). PCR was run as recommended by the manufacturer in a 96.96 Dynamic Array-Gene Expression IFC (Fluidigm Corporation). The relative expression of each gene was quantified by the comparative cycle threshold (Ct) method (ΔΔCt), using *GUSB* as endogenous control and taking as a calibrator the Universal Human Reference RNA (Life Technologies).

### Quantification of CD69 and CD11b levels by flow cytometry

Cell suspensions from PB, LN and BM were washed and blocked with 10% mouse serum (Sigma). Samples were then stained with anti-CD19-FITC (Becton Dickinson), PI, and either anti-CD69-PE or anti-CD11b-PE (Becton Dickinson) and analyzed in an Attune flow cytometer. In each quantification experiment, a constant calibrator sample was stained simultaneously. Expression data was reported as the percentage of viable CLL cells positive for CD69 or CD11b after subtraction of cells stained for IgG1 isotype-PE (Becton Dickinson).

### Co-culture assays

Human bone marrow-derived mesenchymal cell line HS-5 (American Type Culture Collection)(ATCC^®^, CRL­11882™) and human follicular dendritic cell-like cell line HK (kindly provided by Dr. Y.S. Choi) were cultured in Dulbecco's modified Eagle's medium (DMEM, Life Technologies) and Iscove's modified Dulbecco's medium (IMDM, Life Technologies), respectively, and supplemented as above. Before setting up the experiment, HS-5 (2×10^4^ cells) and HK cells (1×10^4^ cells) were plated overnight and, once obtained a confluent stromal monolayer, CLL cells were added at a 1:10 (HS-5:CLL) and 1:20 (HK:CLL) ratio. After 24 hours, CLL cells were analyzed for CD69 expression. At that time, 25 μM bendamustine was added for 24 hours and CLL viability was analyzed by flow cytometry.

### Statistical analysis

Spearman correlation was used to evaluate the dependence of bendamustine cytotoxicity on gene or protein expression. Mann Whitney U Test was used to evaluate response differences among unpaired samples and Wilcoxon Signed Rank Test was used when the samples were paired. These analyses were performed using GraphPad Prism 4.0 software (GraphPad Software).

Multivariate linear regression models were used to determine the relationship between sensitivity to bendamustine and gene, protein and/or clinical variables. Logarithmic transformations were used when necessary to correct for non-normal distributions.

In order to obtain the combination of validated genes that best predicts the response to bendamustine, a linear regression model was fitted for each combination of one to six genes. The RMSE metric was used to compare the performance of each model, which was estimated using the *errorest* function in the *ipred* package of R software with 800 bootstrap samples. Normality of the residuals was checked for the models with lowest RMSE. Lower values of RMSE indicate better fit of the model.

To confirm the independence of candidate predictors from clinical variables, a stepwise linear regression with backward elimination of the non-significant variables was performed. All linear regression models were calculated using R software. Statistical significance was considered when *p*-value < 0.05 (* *P* < 0.05, ** *P* < 0.01, *** *P* < 0.001).

## SUPPLEMENTARY MATERIAL TABLES


