# Supplementary material for: CD69 expression potentially predicts response to bendamustine and its modulation by ibrutinib or idelalisib enhances cytotoxic effect in chronic lymphocytic leukemia
Source: Oncotarget. 2015 Dec 19;7(5):5507–20. doi: 10.18632/oncotarget.6685 (PMC4868702; doi:10.18632/oncotarget.6685)
Supplement: Supplementary file 1 [file oncotarget-07-5507-s001.pdf]

CD69 expression potentially predicts response to bendamustine and its modulation by ibrutinib or idelalisib enhances cytotoxic effect in chronic lymphocytic leukemia

Supplementary Material

Supplemental table 1: Detailed clinical and biological characteristics of CLL patients included in the study.

Patient	Micro arrays cohort (n=38)	qRT-PCR cohort (n=77)	Gender/ Age at diagnosis	a % Tumoral cells	b Binet/ Rai stage	Treatment lines	c IGVH	d ZAP-70	d CD38	d CD49d	e Cyto-genetics	*f Recurrent mutations*	% Cytotoxicity bendamustine 25 µM (24 h)
CLL 01	Y		M/49	90	A/I	0	um	-	-	ND	N	*SF3B1, DAPK1*	46.83
CLL 02	Y		F/59	95	A/0	0	um	+	+	ND	N	*NOTCH1, MGA, CCND2, XPO1*	35.26
CLL 03	Y		M/63	95	C/IV	ND	um	+	+	ND	13q del	*TP53, ASXL1*	31.6
CLL 04	Y	Y	M/44	98	A/I	0	um	-	-	+	13q del	*ZNF292, CNOT3*	12.8
CLL 05	Y	Y	M/70	95	B/II	0	um	-	-	-	13q del	*ATM, SF3B1*	37.5
CLL 06	Y	Y	M/55	96	B/II	0	um	-	-	ND	N	*NOTCH1, RPS15, SETD1A, SETD2*	49.5
CLL 07	Y	Y	M/59	95	A/0	0	ND	+	-	+	N	*ATM, ZNF292, ZMYM3, NFKBIE*	18.2
CLL 08	Y	Y	M/58	88	B/II	0	um	-	-	-	13q del	*BRAF, MGA, ATRX*	79.2
CLL 09	Y	Y	F/52	95	B/II	0	m	-	-	ND	13q del		11.1
CLL 10	Y	Y	M/56	94	A/0	0	um	+	+	+	13q del	*SF3B1, XPO1*	29.8
CLL 11	Y	Y	M/64	93	A/0	0	m	-	-	-	trisomy12	*NF-KB2*(del)	17.9
CLL 12	Y	Y	M/49	94	A/0	0	um	+	+	ND	N	*BRAF, SRSF1*	17.1
CLL 13	Y	Y	M/46	96	A/I	0	um	-	+	-	11q del	*ATM*	24.6
CLL 14	Y	Y	F/77	92	B/II	0	um	+	+	-	11q del, 13q del	*NOTCH, BIRC, ZNF292, MGA*	22.6
CLL 15	Y	Y	M/53	91	A/0	0	m	-	-	-	N		30.5
CLL 16	Y	Y	F/62	96	A/0	0	m	-	-	-	13q del		27.4
CLL 17	Y	Y	M/49	96	C/IV	0	m	-	-	-	13q del	*CHD2, MYD88*	37.9
CLL 18	Y	Y	M/73	95	C/III	0	um	-	+	+	13q del	*ATM, SF3B1, ASXL1*	4.9
CLL 19	Y	Y	M/70	96	C/III	0	um	+	-	-	13q del	*POT1*	22.8
CLL 20	Y	Y	M/71	97	A/0	0	um	-	-	+	N	*FUBP1, XPO1, RPS15*	39.5
CLL 21	Y	Y	M/64	97	A/0	0	m	-	-	-	13q del	*CHD2, SRSF1*	42.7
CLL 22	Y	Y	M/61	96	C/III	0	um	-	-	-	11q del	*BIRC3*(del), *MED12*	22.4
CLL 23	Y	Y	F/68	96	A/0	0	m	-	-	-	13q del	*TLR2*	44.6
CLL 24	Y	Y	F/75	87	B/II	0	um	+	-	+	trisomy12		40.4
CLL 25	Y	Y	M/56	92	A/0	0	m	-	-	-	N		28.5
CLL 26	Y	Y	F/71	83	A/0	0	um	+	-	-	13q del		43.6

CLL 27	Y	Y	M/78	94	A/II	0	um	-	-	+	13q del		23.2
CLL 28	Y	Y	M/60	91	A/0	0	um	+	-	-	N		19.9
CLL 29	Y	Y	F/43	95	A/0	0	m	+	-	-	13q del		44.5
CLL 30	Y	Y	M/44	97	B/II	0	um	-	-	-	13q del	*XPO1*	8.3
CLL 31	Y	Y	F/48	91	A/0	0	m	-	-	-	13q del		42.3
CLL 32	Y	Y	M/78	95	A/0	0	m	-	+	-	trisomy12	*NFKB2*(del)	8.1
CLL 33	Y	Y	M/80	96	A/0	0	m	-	-	-	N		35.1
CLL 34	Y	Y	M/68	93	A/0	0	m	-	-	+	17p del	*TP53*	12.5
CLL 35	Y	Y	F/52	92	B/II	0	m	-	-	ND	trisomy12		42.2
CLL 36	Y	Y	M/54	96	B/II	0	m	+	-	-	13q del		16.8
CLL 37	Y	Y	M/57	98	A/0	0	m	-	+	-	13q del		51.1
CLL 38	Y	Y	M/41	89	A/0	0	m	-	-	-	13q del	*ZFHX3*	30
CLL 39		Y	M/58	95	A/0	0	m	-	-	-	N	*AHNAK, ZMYM3*	19.9
CLL 40		Y	F/66	78	A/0	0	m	-	-	+	13q del		41.8
CLL 41		Y	M/45	95	C/IV	0	m	-	-	ND	13q del	*MYD88, KLHL6*	50.6
CLL 42		Y	M/37	64	A/I	1	m	-	-	ND	13q del	*TP53,CHD2*	9.2
CLL 43		Y	F/53	94	B/II	1	um	+	-	-	13q del, 11q del	*SF3B1, POT1, IKZF3*	20.8
CLL 44		Y	F/60	86	C/III	0	m	+	+	ND	13q del		42.5
CLL 45		Y	M/51	81	A/0	0	m	-	-	ND	13q del, 11q del	*BIRC3, NXFI*	13.3
CLL 46		Y	M/53	97	C/IV	1	um	+	+	+	13q del	*ZNF292, POT1*	0
CLL 47		Y	M/52	97	A/II	2	um	-	+	-	11q del	*ARID1A, ATM*	66.1
CLL 48		Y	M/51	97	B/II	1	um	+	+	-	13q del		27.9
CLL 49		Y	M/52	99	B/I	0	um	+	-	ND	17p del		10.9
CLL 50		Y	F/45	86	B/II	1	um	+	+	+	trisomy12	*NOTCH1, TP53, EGR2, NFX1*	27.7
CLL 51		Y	M/56	99	B/II	4	m	-	+	ND	13q del, 11q del		1.1
CLL 52		Y	M/62	99	C/IV	1	m	-	-	+	13q del		4.7
CLL 53		Y	M/46	93	A/I	1	um	-	+	+	11q del	*ATM*	18.5
CLL 54		Y	M/44	96	C/IV	0	m	-	-	ND	13q del		20.9
CLL 55		Y	M/66	95	C/IV	0	m	-	-	-	13q del		30.5
CLL 56		Y	M/48	92	A/0	0	m	-	-	-	N		38.1
CLL 57		Y	F/44	95	A/0	0	m	-	-	-	N		18.3
CLL 58		Y	M/67	99	A/0	0	m	-	-	-	N	*CHD2*	21.5
CLL 59		Y	M/65	94	A/I	0	um	+	-	-	13q del		12.4
CLL 60		Y	M/68	95	B/II	0	um	-	-	-	13q del	*DDX3X*	0
CLL 61		Y	M/58	93	A/I	0	m	-	-	-	N	*PTPN11*	19.2
CLL 62		Y	F/72	80	A/0	0	m	-	-	+	13q del		40.8
CLL 63		Y	M/56	93	A/0	0	m	-	-	-	N		52.8
CLL 64		Y	M/61	93	A/0	0	m	-	-	-	N		35.1
CLL 65		Y	M/73	72	A/0	0	m	-	-	-	trisomy12	*CCND2*	33.4
CLL 66		Y	F/49	94	A/I	0	m	-	-	-	13q del		27.8

CLL 67	Y	F/49	ND	A/I	0	m	ND	ND	ND	13q del		48
CLL 68	Y	M/66	94	A/0	0	m	-	-	-	trisomy12, 13q del	*CHD2, KLHL6*	22.6
CLL 69	Y	M/62	97	B/II	0	um	-	-	-	13q del, 11q del	*BIRC3*(del)*, ATR*	56.5
CLL 70	Y	M/63	97	A/I	2	um	+	-	ND	13q del		5.5
CLL 71	Y	M/63	85	A/0	0	m	-	-	-	N		41
CLL 72	Y	M/69	98	A/0	0	m	-	-	-	N		47.9
CLL 73	Y	F/69	98	A/0	1	m	-	-	-	N	*CHD2*	18.5
CLL 74	Y	F/52	85	A/0	0	m	-	-	ND	trisomy12		23.3
CLL 75	Y	M/54	95	A/I	0	m	+	-	ND	13q del		8.3
CLL 76	Y	M/62	95	A/0	0	um	+	+	ND	N	*ATM, NOTCH, BIRC3, CHD2, MGA*	42.7
CLL 77	Y	M/66	98	B/III	0	um	-	+	-	11q del	*ATM, BIRC3*(del)	3.1
CLL 78	Y	F/65	86	A/0	0	m	-	-	-	13q del		37.3
CLL 79	Y	M/67	94	A/0	0	m	-	-	-	13q del	*CNOT3*	7.1
CLL 80	Y	F/54	92	B/II	0	m	-	-	+	N		14.1

Abbreviations: Y, yes; M, male; F, female; ND, not determined m, mutated; um, unmutated; N, normal; del, deletion; BDM, bendamustine.
a Percentage of tumoral cells was quantified by flow cytometry labeling of CD5+/CD19+ cells and light chain restriction.
b According to Rai and Binet's classification: Early (Rai 0, Binet A), intermediate (Rai I/II, Binet B) and advanced (Rai III/IV, Binet C) stage disease.
c IGVH gene was sequenced after PCR amplification, and aligned to NCBI IgBlast. Mutated status was assigned when >2% deviation from germline IGVH sequence was present.
d Expression levels of ZAP-70, CD38 and CD49d were determined by flow cytometry with conjugated antibodies. Cut-off considered for positivity was >20%, >30% and >30%, respectively.
e Cytogenetic alterations were assessed by FISH.
f Recurrent mutations were identified by exome-sequencing analysis within the ICGC project.

Supplemental table 2: List of DEGs between bendamustine-resistant and bendamustine-sensitive CLL cases obtained by a Rank Products analysis using an FDR<0.001 and a FC >1.5.

Downregulated in resistant cases

Gene	Fold-change BDM-Resistant vs BDM-Sensitive	Gene	Fold-change BDM-Resistant vs BDM-Sensitive	Gene	Fold-change BDM-Resistant vs BDM-Sensitive	Gene	Fold-change BDM-Resistant vs BDM-Sensitive	Gene	Fold-change BDM-Resistant vs BDM-Sensitive
NTN3	-1.504	*CTLA4*	-1.657	*HSPA1A*	-1.760	*CCDC88A*	-1.837	*SYTL2*	-1.901
C5orf32	-1.511	*HSBP1L1*	-1.661	*KCTD7*	-1.761	*SLAMF1*	-1.838	*UTS2*	-1.903
LY86	-1.512	*ANK1*	-1.665	*GLUL*	-1.772	*SIPA1L1*	-1.839	*DEF8*	-1.907
CREM	-1.528	*SATB1*	-1.670	*ATM*	-1.773	*RASGRP1*	-1.844	*RAD51L1*	-1.908
C21orf7	-1.544	*PRPF4*	-1.674	*CCNG2*	-1.775	*PPP4R2*	-1.854	*ATF5*	-1.911
KLF7	-1.547	*DUSP22*	-1.675	*GOLPH3L*	-1.779	*RAD51C*	-1.856	*SELP*	-1.913
MAP3K8	-1.551	*HSPA1A*	-1.675	*NCRNA00171*	-1.788	*PAIP2B*	-1.858	*HERC4*	-1.914
EGR3	-1.554	*ADARB1*	-1.676	*OSGEPL1*	-1.788	*ANKFY1*	-1.860	*GSPT1*	-1.915
CD86	-1.570	*VPS37B*	-1.686	*COL4A3*	-1.789	*C11orf61*	-1.860	*FBXO15*	-1.918
SERPINB6	-1.585	*ESYT2*	-1.690	*SLC37A4*	-1.790	*KLHDC5*	-1.860	*GLYATL2*	-1.919
COBLL1	-1.590	*IGLL5*	-1.700	*CD22*	-1.791	*PHF23*	-1.861	*ZNF594*	-1.920
SYNJ2	-1.596	*PPP1R10*	-1.704	*SLC4A7*	-1.792	*C18orf1*	-1.863	*TUBB1*	-1.921
IGF2BP3	-1.606	*MAP4K3*	-1.705	*SKI*	-1.795	*TBXA2R*	-1.864	*IL2RB*	-1.922
HIST1H2BD	-1.611	*PTCH1*	-1.708	*BCL2*	-1.801	*TCTN1*	-1.864	*SPIN4*	-1.923
SLC7A1	-1.611	*ZNF429*	-1.709	*LEPROTL1*	-1.804	*PGBD2*	-1.865	*FAM65B*	-1.926
RHOBTB2	-1.615	*ANXA4*	-1.727	*TTC33*	-1.806	*TSHZ2*	-1.868	*GPT2*	-1.926
TRIM7	-1.616	*BCAR3*	-1.739	*SOS1*	-1.811	*NFATC1*	-1.869	*SGK223*	-1.927
ZBTB1	-1.618	*BATF*	-1.740	*SLAMF6*	-1.815	*TAF4*	-1.872	*PTPRS*	-1.929
ZADH2	-1.628	*PPP2CB*	-1.742	*ZNF404*	-1.820	*ZFP3*	-1.876	*IGJ*	-1.931
WNT3	-1.631	*SH3PXD2A*	-1.747	*SMA4*	-1.821	*HSPH1*	-1.878	*SLCO4A1*	-1.932
PYHIN1	-1.632	*ANKRD20A5*	-1.747	*SLC16A3*	-1.823	*HOMER3*	-1.885	*C17orf87*	-1.934
PNMA2	-1.642	*RAPGEF5*	-1.749	*APBB2*	-1.826	*PLGLA*	-1.887	*PDCD6IP*	-1.937
SESTD1	-1.643	*MRC2*	-1.752	*ELP3*	-1.832	*C20orf195*	-1.890	*C2orf88*	-1.937
HSPA1B	-1.650	*POPDC2*	-1.757	*STK38*	-1.837	*PAQR8*	-1.892	*TRAK2*	-1.938
MAP2K6	-1.650	*TTC28*	-1.757	*SLC35B3*	-1.837	*FCER2*	-1.896	*NBPF1*	-1.939
MAP4K4	-1.657	*FGFR1*	-1.758	*COL4A4*	-1.837	*ZNF232*	-1.899	*C10orf26*	-1.942

Gene	Fold-change BDM-Resistant vs BDM-Sensitive	Gene	Fold-change BDM-Resistant vs BDM-Sensitive	Gene	Fold-change BDM-Resistant vs BDM-Sensitive	Gene	Fold-change BDM-Resistant vs BDM-Sensitive
PDGFD	-1.944	*PTH2R*	-2.069	*SIK2*	-2.275	*UBASH3B*	-2.480
PITPNC1	-1.946	*ARMCX2*	-2.069	*CDC14B*	-2.277	*COL9A3*	-2.487
C3orf26	-1.947	*TCL1A*	-2.072	*PRDM15*	-2.279	*TXNDC5*	-2.500
ITPKB	-1.957	*ZNF540*	-2.077	*PDE8A*	-2.279	*CRIP3*	-2.524
DIDO1	-1.958	*KCNH8*	-2.081	*IL4R*	-2.283	*RASSF6*	-2.549
SLC15A2	-1.960	*KRT18*	-2.088	*RFTN1*	-2.284	*FCRL2*	-2.583
ITGB1	-1.962	*VIPR1*	-2.096	*PDE4A*	-2.301	*HLA-DRB4*	-2.608
TOR1B	-1.964	*UBN2*	-2.103	*KLHL6*	-2.311	*SOBP*	-2.623
KIAA0649	-1.972	*SEC63*	-2.107	*TCF4*	-2.331	*LHFPL2*	-2.629
SYNE2	-1.975	*RNF144B*	-2.129	*NAPEPLD*	-2.340	*PTK2*	-2.646
C17orf80	-1.984	*SLC4A8*	-2.137	*USP44*	-2.341	*SYT17*	-2.664
APPBP2	-1.987	*VASH1*	-2.144	*PPBP*	-2.353	*MYBL1*	-2.689
PIGR	-1.995	*TRD@*	-2.145	*SEMA4A*	-2.361	*TRAC*	-2.720
CD200	-1.996	*PROSC*	-2.147	*FMOD*	-2.365	*PPAPDC1B*	-2.871
ROCK2	-1.997	*DPYSL2*	-2.148	*LCK*	-2.374	*FCRL1*	-2.930
ZCCHC18	-2.000	*PASK*	-2.152	*HOXB2*	-2.376	*NBPF3*	-2.965
EPM2AIP1	-2.012	*DNAJB6*	-2.177	*PGM2L1*	-2.376	*FCRL5*	-2.980
FILIP1L	-2.021	*LRRC32*	-2.179	*BACE2*	-2.381	*TGFBI*	-2.995
ZNF137P	-2.024	*WNT10A*	-2.220	*APOD*	-2.381	*XIST*	-3.442
SLC25A19	-2.028	*ORMDL1*	-2.220	*MEF2C*	-2.396	*KIAA0802*	-3.834
ANKH	-2.031	*ATP1B1*	-2.222	*TNFRSF17*	-2.397	*SDK2*	-3.857
QSER1	-2.041	*C11orf73*	-2.223	*GTF2H1*	-2.406	*P2RY14*	-4.171
DHX32	-2.041	*IKZF2*	-2.234	*ADAM29*	-2.429	*COCH*	-4.358
HVCN1	-2.042	*PLGLB2*	-2.244	*ATP10D*	-2.430	*CNTNAP2*	-4.564
PF4	-2.063	*PLGLB1*	-2.252	*PTPLA*	-2.457	*TCF7*	-4.733
LPAR5	-2.064	*GTSF1L*	-2.268	*HRK*	-2.467	*FCRL3*	-4.873
C5orf53	-2.068	*PHTF1*	-2.270	*TREML2*	-2.467	*HLA-DPB1*	-5.342

Upregulated in resistant cases

Gene	Fold-change BDM-Resistant vs BDM-Sensitive	Gene	Fold-change BDM-Resistant vs BDM-Sensitive	Gene	Fold-change BDM-Resistant vs BDM-Sensitive	Gene	Fold-change BDM-Resistant vs BDM-Sensitive	Gene	Fold-change BDM-Resistant vs BDM-Sensitive
LYZ	9.553	*RGS1*	2.762	*RORA*	2.220	*STAT4*	2.039	*CEBPD*	1.883
S100A8	7.514	*CYorf15B*	2.745	*CCL3*	2.217	*TNFRSF13B*	2.038	*WIPF1*	1.868
S100A9	5.511	*ITGA4*	2.725	*YPEL2*	2.211	*TSPAN14*	2.007	*RPGR*	1.860
HLA-C	5.360	*PTPRJ*	2.708	*UTY*	2.207	*DMD*	2.005	*ARRDC3*	1.858
DDX3Y	4.589	*CASP8*	2.635	*SERPINF1*	2.200	*TNFSF9*	1.991	*HOOK1*	1.847
RPS4Y1	4.508	*GPR183*	2.621	*ZNF608*	2.148	*SNX5*	1.988	*IL8*	1.845
HLA-DQA1	4.207	*IFI44*	2.590	*ITGAM*	2.143	*ANKRD36B*	1.984	*GPM6A*	1.839
MYADM	4.018	*GNB4*	2.481	*TAGAP*	2.138	*LRRK2*	1.981	*BCL7A*	1.833
HLA-DRB5	3.966	*DIP2C*	2.453	*GAS7*	2.133	*ZBTB43*	1.979	*NDST1*	1.824
TNS3	3.942	*LOC728741*	2.450	*C1orf228*	2.132	*ID2*	1.973	*C1orf162*	1.823
KLF10	3.937	*HLA-DPB1*	2.448	*BCL2A1*	2.122	*IL15*	1.971	*DDIT3*	1.822
EGR1	3.860	*CLEC2B*	2.442	*VCAN*	2.121	*MAFB*	1.968	*CNTLN*	1.815
KLRK1	3.859	*ZNF300*	2.427	*RNF145*	2.117	*TPM3*	1.966	*ZBP1*	1.803
HLA-DQB1	3.857	*DUSP1*	2.408	*FOSB*	2.105	*APP*	1.961	*TMEM56*	1.792
ANXA1	3.784	*DUSP6*	2.404	*FKBP5*	2.103	*AIF1*	1.959	*ETV3*	1.789
KIAA1324L	3.580	*C1orf38*	2.385	*SGCE*	2.090	*YES1*	1.942	*STK17B*	1.789
CCL4	3.467	*ITGAL*	2.370	*DUSP26*	2.079	*DPYD*	1.932	*DUSP10*	1.788
ZNF711	3.149	*RBMS3*	2.368	*SPTBN1*	2.070	*G0S2*	1.930	*CYorf15A*	1.786
FOS	3.137	*CD1C*	2.361	*ENPP2*	2.067	*DDIT4*	1.922	*TES*	1.784
FCN1	3.071	*DUSP5*	2.309	*TRPS1*	2.065	*OSBPL3*	1.908	*PPIG*	1.783
LGALS3	3.051	*USP9Y*	2.305	*PELI1*	2.056	*KLF6*	1.905	*AIM1*	1.780
AHR	3.024	*SGK1*	2.298	*CRY1*	2.055	*CRYM*	1.901	*RTN4*	1.768
TFEC	2.999	*CD69*	2.285	*RGS2*	2.053	*BTG1*	1.894	*C6orf114*	1.767
KDM5D	2.976	*MYC*	2.285	*IL1B*	2.051	*KLK1*	1.894	*C17orf91*	1.766
PSD3	2.831	*IFI44L*	2.281	*GADD45B*	2.049	*BCL2*	1.891	*TIMP1*	1.763
EIF1AY	2.803	*IREB2*	2.222	*EML4*	2.042	*PTPRG*	1.886		

Supplemental table 3: List of DEGs between bendamustine-resistant and bendamustine-sensitive CLL cases classified according to the biologic functions encountered by IPA.

Cellular movement		Cell-to-cell signaling and interaction		Cellular development			Cellular growth and proliferation			Cell death and survival		
ADARB1	MYADM	AHR	PPBP	AHR	IGF2BP3	RORA	ADARB1	FOS	PTK2	AHR	ID2	PTPRG
AIF1	MYC	ANXA1	PTK2	AIF1	IL15	SATB1	AHR	GADD45B	PTPRG	ANXA1	IGF1R	RAD51C
ANXA1	NFATC1	ATM	PTPRJ	ANXA1	IL1B	SERPINF1	AIF1	GLUL	PTPRJ	ANXA4	IGF2BP3	RHOB
APP	PF4	BCL2A1	RASGRP1	APP	IL2RB	SERTAD1	ANXA1	HSPA1A/HSPA1B	PTPRS	APP	IL15	RHOBTB2
ARRDC3	PIGR	CCL3	RGS1	ARRDC3	IL4R	SGK1	APP	ID2	PYHIN1	ATM	IL1B	RNF144B
BCAR3	PON2	CD1C	RHOB	ATF5	IL6ST	SH3PXD2A	ARRDC3	IGF1R	RHOB	BCL2A1	IL2RB	RNF41
BCL2	PPBP	CD200	ROCK2	ATM	IL8	SKI	ATF5	IGF2BP3	RHOBTB2	BTG1	IL4R	ROCK2
CCDC88A	PTK2	CD86	S100A8	BATF	ITGAM	SOS1	ATM	IL15	RNF41	CAMK2D	IL6ST	RTN4
CCL3	PTPRJ	COL4A3	S100A9	BCAR3	ITGB1	SSBP2	BATF	IL1B	RORA	CASP8	IL8	S100A8
CD86	RAB11FIP1	CTLA4	SELP	BTG1	JUN	STAT4	BCAR3	IL2RB	SATB1	CCL3	ITGAL	S100A9
COL4A3	RASGRP1	DDIT4	SERPINF1	CASP8	KLF10	STK38	BCAT1	IL4R	SERPINF1	CD22	ITGAM	SATB1
CRY1	RGS1	EGR3	SH3PXD2A	CCL3	KLF4	TCF4	BCL2	IL6ST	SERTAD1	CD69	ITGB1	SERPINF1
DIDO1	RHOB	FAM65B	TCL1A	CCNG2	KLF6	TCL1A	BCL2A1	IL8	SGK1	CD70	JUN	SGK1
DNAJB6	RNF41	FCER2	TGFBI	CD70	KLRK1	TES	BTG1	ITGA4	SKI	CEBPD	KANK2	SH3PXD2A
DPYSL2	ROCK2	FCGR2A	TIMP1	CD86	LCK	TIMP1	CASP8	ITGB1	SLAMF1	COL4A3	KLF4	SLAMF6
EGR1	RTN4	FGFR1	TNFRSF13B	CEBPD	LGALS3	TNFRSF13B	CCL3	JUN	SOS1	CREM	KLF6	SLC37A4
FAM65B	S100A8	FOS	TNFRSF17	COL4A3	LRRK2	TNFRSF17	CCNG2	KANK2	STK38	CTLA4	KLRK1	SLC7A1
FGFR1	S100A9	HSPH1	TNFSF9	CREM	LST1	TNFSF9	CD70	KLF10	TCF4	DDIT3	KRT18	STK17B
FOS	SATB1	IGF1R	TYROBP	CTLA4	MAFB	UTS2	CD86	KLF4	TCL1A	DDIT4	LCK	STK38
ID2	SELP	IL15	VCAN	DDIT3	MAP2K6	VASH1	CEBPD	KLF6	TES	DIDO1	LGALS3	SYF2
IGF1R	SEMA4A	IL1B	VIPR1	DIDO1	MAP4K4	VCAN	CLK1	KLRK1	TGFBI	DPYD	LRRK2	TCF4
IL15	SERPINF1	IL2RB		DMD	MEF2C		COL4A3	LCK	TIMP1	DUSP1	LY86	TCL1A
IL1B	SH3PXD2A	IL4R		DUSP1	MYC		CREM	LGALS3	TNFRSF13B	DUSP10	LYZ	TIMP1
IL8	SKI	ITGA4		DUSP5	NFATC1		CTLA4	LRRK2	TNFRSF17	DUSP22	MAP2K6	TNFRSF13B
ITGA4	SOS1	ITGAL		DUSP6	NR4A2		DDIT3	LST1	TNFSF9	DUSP5	MAP3K8	TNFRSF17
ITGAL	TBXA2R	ITGAM		EGR1	PDCD6IP		DIDO1	LY86	UTS2	DUSP6	MEF2C	TNFSF9
ITGAM	TGFBI	ITGB1		EGR3	PF4		DUSP1	MAP2K6	VASH1	EGR1	MYC	TRPS1
ITGB1	TIMP1	KLRK1		FCER2	PPP1R10		DUSP22	MAP4K4	VCAN	EGR3	NFATC1	WIPF1
JUN	TNFSF9	LCK		FGFR1	PTCH1		DUSP5	MYC	VIPR1	FCER2	NR4A2	
KLF4	TNS3	LGALS3		FKBP5	PTK2		DUSP6	NFATC1	WIPF1	FEM1B	PDCD6IP	
KLRC4-KLRK1/KLRK1	TPM3	MAP3K8		FOS	PTPRJ		EGR1	NR4A2		FGFR1	PF4	
LCK	UTS2	MYADM		GADD45B	PYHIN1		EGR3	PDCD6IP		FKBP5	PIGR	
LGALS3	VASH1	MYC		GAS7	RHOB		EIF1AY	PF4		FOS	PPBP	
MAP2K6	VCAN	PDCD6IP		HSPA1A	RHOBTB2		FCER2	PPP1R10		G0S2	PPP1R10	

MAP3K8	*VIPR1*	*PF4*		*ID2*	*RNF41*		*FGFR1*	*PPP1R15A*		*GADD45B*	*PPP1R15A*
MAP4K4		*PIGR*		*IGF1R*	*ROCK2*		*FKBP5*	*PTCH1*		*HSPA1A*	*PTK2*

Supplemental Table 4. List of genes analyzed for validation by qRT-PCR. 46 genes were selected for its role in the top biological functions encountered by IPA analysis.

Gene	p-value
AIF1	**P*=0.0148
ANXA1	*P*=0.6540
APP	**P*=0.015
BCL2A1	***P*=0.0047
CCL3	*P*=0.1890
CCL4	*P*=0.3280
CD200	*P*=0.5470
CD69	****P*=0.0002
CRY1	**P*=0.0311
DMD	*P*=0.3890
DNAJB6	*P*=0.5581
DUSP1	****P*=0.0004
DUSP5	**P*=0.0320
EGR1	**P*=0.0198
FOS	***P*=0.0048
GADD45	**P*=0.0283
GAS7	*P*=0.471
HRK	*P*=0.228
ID2	*P*=0.714
IL15	*P*=0.0602
IL1B	*P*=0.8460
ITGA4	*P*=0.3740
ITGAL	*P*=0.1101
ITGAM	***P*=0.0016
KLRC4	*P*=0.6260
LCK	*P*=0.0594
LGALS3	**P*=0.0176
LRRK2	*P*=0.3650
MAFB	*P*=0.8370
MEF2C	*P*=0.2930
MYC	*P*=0.0838
PF4	*P*=0.4104
PPBP	*P*=0.5990
PTK2	*P*=0.2051
RNF144B	*P*=0.2220
ROCK2	*P*=0.8904
S100A8	*P*=0.5390
S100A9	*P*=0.4390
SERPINF1	*P*=0.1350
SGK1	*P*=0.2470
STAT4	*P*=0.1075
TCL1A	*P*=0.9051
TNFRSF13B	*P*=0.0680
TNFSF9	**P*=0.0138
VASH1	*P*=0.1840
VCAN	*P*=0.8007

*, P<0.05; **, P<0.01; ***, P<0.001.
